# Survival after multiple in-hospital cardiac arrests due to severe amitriptyline poisoning– a case report

**DOI:** 10.1186/s12245-025-01109-6

**Published:** 2026-01-03

**Authors:** Amanuel Dagabas Wakoya, Tadesse G. Asenabeto, Hawi D. Moti, Negash B. Bayu, Fenta Wondimneh, Abebe D. Ayinalem, Alemu B. Mesekere, Ayto A. Negash, Tiliksew A. Tesfaw

**Affiliations:** 1 Department of Emergency and Critical Care Medicine, Haramaya university, Harar, Ethiopia; 2 School of Nursing, Haramaya university, Harar, Ethiopia; 3 Department of Emergency Medicine and Critical Care, Worabe Comprehensive Specialized Hospital, Werabe University, Central Ethiopia, Ethiopia

**Keywords:** Severe amitriptyline toxicity, Multiple cardiac arrest, ACLS, Refractory arrhythmias, Sodium bicarbonate

## Abstract

**Background:**

Amitriptyline, a classic tricyclic antidepressant, can produce toxic effects ranging from mild antimuscarinic symptoms to severe cardiotoxicity due to sodium channel blockade. In severe cases, toxicity may rapidly progress to cardiac arrest if not promptly recognized and managed.

**Case presentation:**

We present a 25-year-old man who attempted suicide by ingesting 3.25 g of amitriptyline. He developed two episodes of cardiac arrest and recurrent life-threatening arrhythmias but was resuscitated and later discharged without neurological deficits. Management included aggressive supportive care, administration of sodium bicarbonate as the main antidotal therapy, and prolonged resuscitative efforts in the intensive care unit.

**Conclusion:**

This case highlights that survival with good neurological outcome is possible even after multiple toxin-induced cardiac arrests when timely and persistent resuscitative measures are undertaken. It emphasizes the importance of early recognition of tricyclic antidepressant toxicity, prompt initiation of specific therapy, and preparedness for cardiac complications—particularly in low-resource settings. In addition, this report draws attention to the public health concern related to the accessibility and misuse of tricyclic antidepressants.

## Introduction

Amitriptyline is a tricyclic antidepressant (TCA) belonging to the class of tertiary amine compounds. Introduced in the 1950s, tricyclic antidepressants were among the first generation of drugs developed for the treatment of depression. However, their use has markedly declined over the years with the advent of safer and more selective antidepressants. Despite this, TCAs remain clinically relevant, both for depression and certain chronic pain syndromes. Tricyclic antidepressants are associated with a high risk of toxicity, exhibiting the highest ratio of deaths to exposures among antidepressant overdoses reported to U.S. Poison Control Centers [1]. In 2021, more than one million people in the United States were using amitriptyline, with 4,654 reported poisoning cases, including five deaths — the highest number among TCAs [1, 2].

TCA toxicity typically manifests with hypotension, cardiac dysrhythmias, central nervous system (CNS) depression, and seizures, which can progress to cardiac arrest if not promptly recognized and managed. The most common cause of death following TCA overdose is myocardial depression leading to refractory hypotension, and fatal dysrhythmias like ventricular tachycardia, or ventricular fibrillation. Mortality can reach up to 70% among patients who do not receive timely medical intervention [3]. The cornerstone of management includes airway protection, vasopressor support for hypotension, and intravenous sodium bicarbonate therapy for metabolic acidosis and cardiotoxicity [4]. Here, we present the case of a 25-year-old male with severe amitriptyline poisoning who presented in coma and had two episodes of cardiac arrest and multiple arrhythmias in the emergency department. He was successfully resuscitated and managed with intravenous fluids, vasopressor, mechanical ventilation for three days, and sodium bicarbonate therapy, ultimately making a full neurological recovery and being discharged home in stable condition. We present this case report in accordance with established CARE guidelines [5].

### Case presentation

A 25-year-old Ethiopian man was brought to the emergency department after being found unconscious in his locked room by family members approximately four hours after last being seen. Thirteen empty strips, each containing ten tablets of amitriptyline 25 mg (totaling 3,250 mg) were found beside him, which his family brought with them to the hospital. On arrival, his pulse rate was 155 beats per minute, blood pressure was unrecordable, oxygen saturation was 74% on room air, random blood sugar was 105 mg/dl, and Glasgow Coma Scale (GCS) was 3/15.

**Fig. 1:**
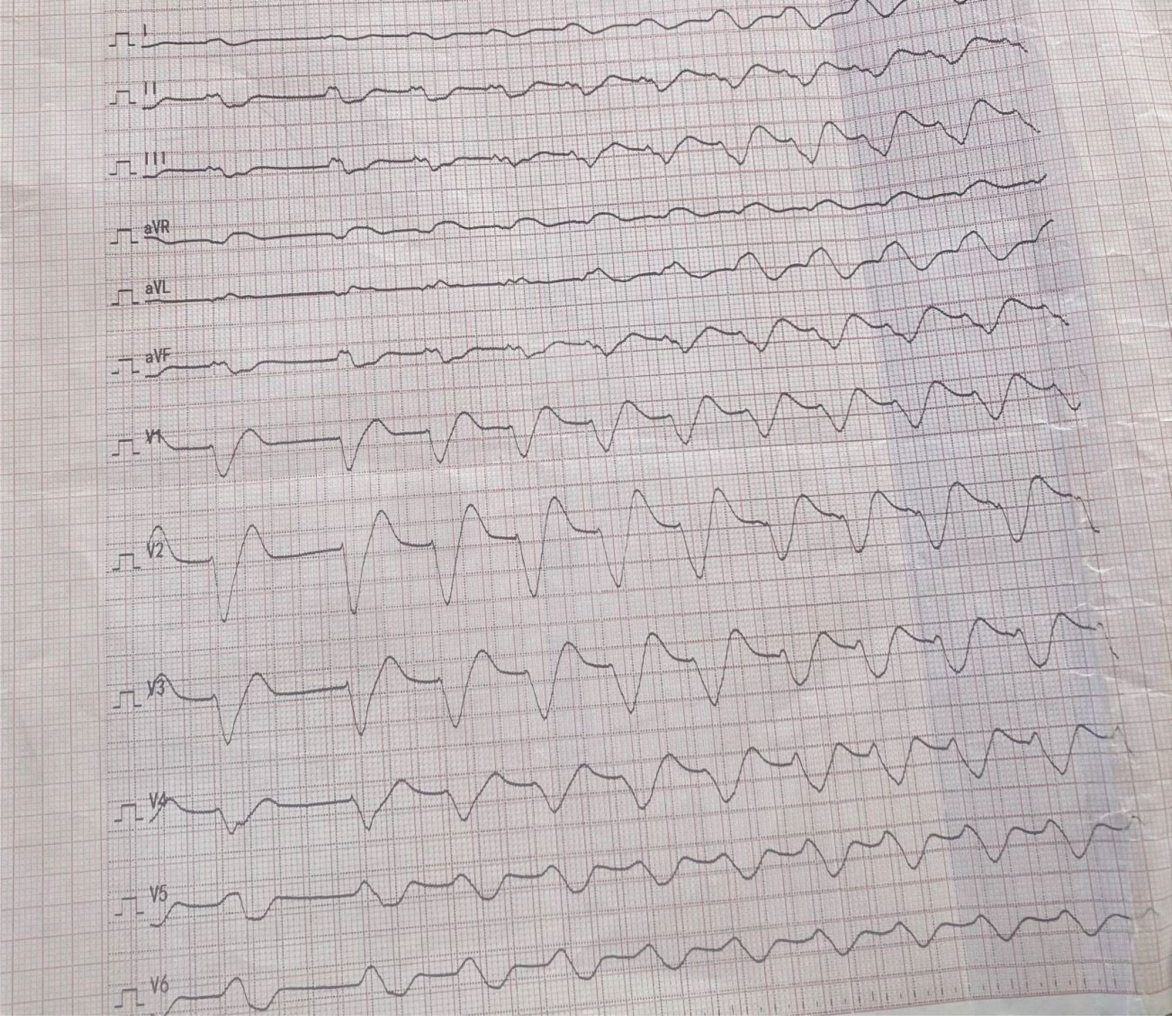
Initial ECG of the patient at emergency showing wide complex tachycardia with QRS duration > 160 ms, and right axis deviation of terminal R wave in lead Avr

He was immediately transferred to the resuscitation area, where an oropharyngeal airway was inserted followed by endotracheal intubation and mechanical ventilation, improving oxygen saturation to 95%. Two large-bore intravenous lines were established, and 2 L of normal saline were rapidly infused. Continuous cardiac monitoring revealed a monomorphic wide-complex ventricular tachycardia at a rate of 158 beats per minute, confirmed on a 12-lead Electrocardiogram (ECG) (Fig. 1). Synchronized cardioversion was done three times (with 100 J,200 J and 360 J respectively) and it was not successful.

**Fig. 2:**
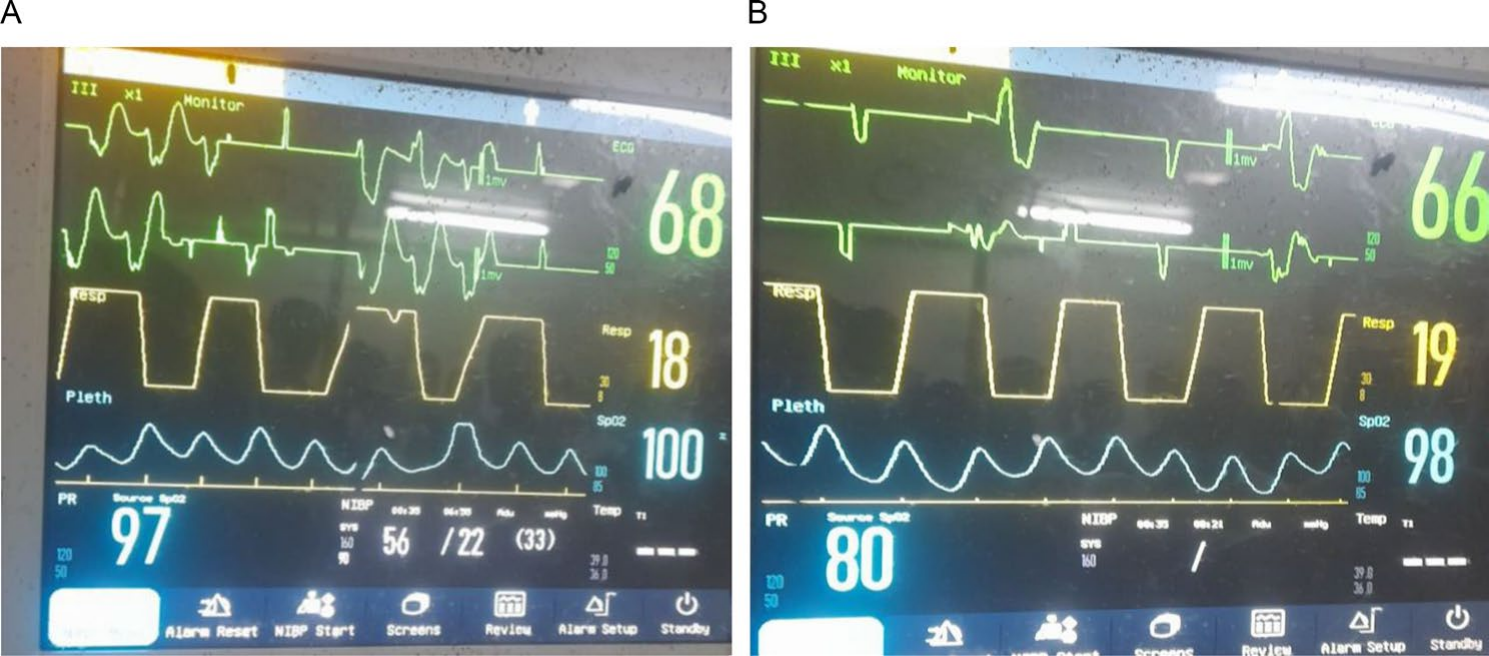
**A** ECG tracing on monitor after initial ROSC showing persistent wide complex rythm. **B** ECG tracing on monitor after the second ROSC

With a diagnosis of severe amitriptyline poisoning with refractory ventricular tachycardia, sodium bicarbonate was planned as an antidote; however, it was unavailable locally and had to be sourced from a pharmacy a nearby city. After 10 min, no central pulse was palpable, and cardiopulmonary resuscitation (CPR) was initiated with Advanced Cardiac Life Support (ACLS) protocol for pulseless ventricular tachycardia. Return of spontaneous circulation (ROSC) was achieved after four cycles of CPR and three defibrillations with 200 J, 250 J and 360 J respectively and two 1 mg IV epinephrine was given. Post-ROSC monitor showed persistent wide complex rhythm (Fig. 2A). Blood pressure also remains low (Fig. 2A) and the patient developed brief generalized seizure for which another 5 mg IV diazepam was given.

While awaiting the antidote, supportive care was continued, including vasopressor support (noradrenaline drip with 0.05mcg/kg/min to be escalated every 15 min), intravenous fluids, mechanical ventilation. Despite these measures, hemodynamic instability and recurrent ventricular tachycardia persisted, another synchronized cardioversion was tried but was not successful. Hypotension also persisted despite escalating noradrenaline to 0.2mcg/kg/min. Mean while the patient had another cardiac arrest with the arrest rhythm being ventricular fibrillation. ROSC was achieved after the third cycle of CPR and two defibrillation with 360 J and the rhythm on monitor after the second ROSC showed PVCs with ventricular conduction delay (Fig. 2B). Upon arrival of sodium bicarbonate, 100 mEq diluted in 100 mL of dextrose 5% water(D5W) was administered two times, and followed by a continuous infusion by adding 150 meq of sodium bicarbonate in 1 L of D5W at a rate of 200 ml/hr.

**Fig. 3:**
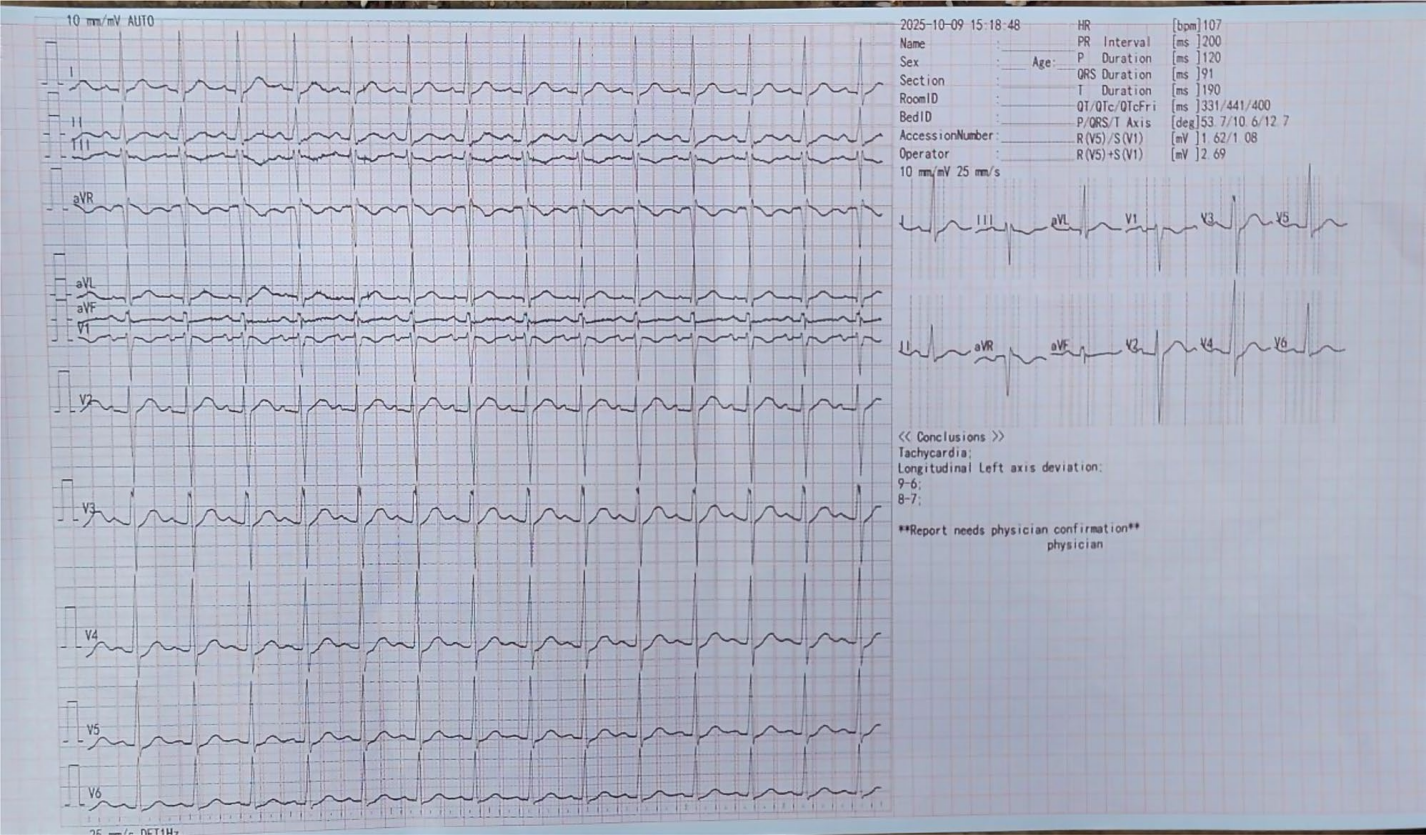
ECG tracing of the patient with amitriptyline poisoning on third day in the ICU after sodium bicarbonate therapy

The patient was transferred to the intensive care unit (ICU), where the same management was continued. His cardiac monitor intermittently showed narrow complex rhythm with intermittent premature ventricular contractions. By the following day, blood pressure stabilized, and the rhythm converted to a narrow-complex tachycardia (maximum rate reaching 126 bpm). His GCS improved to 10T, and gradual weaning from mechanical ventilation was initiated. Sodium bicarbonate infusion was discontinued on the third day after rhythm normalization (Fig. 3) and hemodynamic stability was achieved. Blood gas analysis was not available in our institution. Serial serum electrolyte measurements remained within the normal reference range (Table 1).

He was extubated on the third day, with GCS of 15, and was discharged from the ICU on the fifth day. Upon further inquiry, he had no history of drug use or known medical conditions such as diabetes mellitus or hypertension. However, he had recently been experiencing significant distress and social withdrawal after failing to secure employment since his graduation. The patient was subsequently referred to the psychiatry unit for further evaluation and counseling.

**Table 1:** Investigation summary of the patient

Test	10/6/2025	11/6/2025	12/6/2025	Reference Range and unit
**CBC**				
WBC	16.8		12.3	4000–11,000
Neutrophil	92.7		44.3	55–75%
Lymphocyte	4.3		4.1	25–40%
Monocyte	2.5		2.1	2–6%
Eosinophil	0.2		0.2	1–3%
RBC	5.3		5.6	4-5 × 10^12/L
Hematocrit	44.6		47	36–46%
Hemoglobin	16.4		15.8	12-16gm/dl
MCV	83.6		81.9	800-100 fl.
MCH	30.7		30.6	27-33Pg
MCHC	36.8		34.2	32-36gm/dl
RDW	13.6		14.1	< 14.5%
Platelet	232		255	150-450 × 10^6/L
**Electrolytes**				
Pottasium	4.48	3.80	3.56	3.5-5.3mmol
Sodium	135.4	143	144	137-147mmol
Chloride	103	106	107.9	99-110mmol/L
Ionized calcium	1.04			1.05–1.35 mmol/L
High sensitive cardiac troponin		21.4 pg/ml		0–29 pg/ml
**Liver Function test**				
Alp	80u/L			60–120
ALT	15.6			0–45
AST	28			0–35
AST/ALT	1.8			
Direct Billirubin	0.13			0-0.3
Indirect Billirubin	0.22			0.2–1.2
Total Billirubin	1.59			

## Discussion

Poisoning is a major cause of mortality, particularly among young adults, and represents the leading cause of non-traumatic cardiac arrest in individuals under 35 years. Unlike most cardiac arrest cases, prolonged resuscitation in poisoned patients is often not futile; neurologically intact survival has been reported even after over 60 min of pulseless arrest [6, 7].

Tricyclic antidepressants (TCAs), particularly amitriptyline, are associated with significant cardiotoxicity and fatal arrhythmias. Amitriptyline is highly lipophilic, with a large volume of distribution, and exerts toxicity through multiple mechanisms: inhibition of presynaptic norepinephrine and serotonin reuptake, blockade of cardiac sodium and potassium channels, antagonism of muscarinic acetylcholine receptors, peripheral alpha-1 adrenergic and histamine (H1) receptor blockade, and inhibition of central GABA-A receptors [6, 8].

Clinical manifestations range from mild antimuscarinic effects to life-threatening cardiotoxicity secondary to sodium channel blockade. Common findings include altered mental status, seizures, ileus, dry mouth, urinary retention, hypotension, refractory arrhythmias, and respiratory depression. Severe toxicity may result in secondary complications such as aspiration pneumonia, pulmonary edema, anoxic encephalopathy, hyperthermia, or rhabdomyolysis [1, 9]. Cardiovascular toxicity is the major determinant of mortality. Tachycardia, the most common symptom, arises from anticholinergic effects and norepinephrine reuptake inhibition. Intraventricular conduction delays and QT prolongation, caused by sodium and potassium channel blockade, predispose patients to ventricular tachycardia and cardiac arrest [10]. ECG is essential for risk stratification, guiding therapy, and predicting neurological and cardiovascular complications, often more reliably than serum drug levels [6, 11].

Management of severe amitriptyline poisoning includes supportive care, gastrointestinal decontamination (if early), antidotal therapy with sodium bicarbonate, intravenous fluids, vasopressors, seizure control with benzodiazepines, lipid emulsion therapy, and correction of electrolyte abnormalities. In cardiac arrest, ACLS protocols are applied with modifications; Class IA, IC, and III antiarrhythmic agents are contraindicated due to proarrhythmic risk [12, 13]. Sodium bicarbonate remains the mainstay of therapy, increasing sodium channel gradient, promoting TCA dissociation from channels through alkalinization, and enhancing protein binding to reduce free drug concentration [9]. Vasopressors are used when hypotension persists despite fluids and bicarbonate, and lipid emulsion therapy may be considered for refractory cardiotoxicity [9, 14].

In our patient, delayed presentation precluded gastrointestinal decontamination. Rapid sequence intubation was performed, mechanical ventilation initiated, and diazepam administered for seizure control. Multiple episodes of cardiac arrest were managed according to ACLS, with eventual administration of sodium bicarbonate once available. ABG analysis was not possible; therapy was guided by ECG and hemodynamic response. Sodium bicarbonate was discontinued once rhythm normalized and shock resolved. Lipid emulsion therapy, though potentially beneficial, was unavailable.

In our review of similar cases we identified a 13-year-old female recovered from amitriptyline-induced cardiac arrest with standard care and lipid therapy [2], and a 25-year-old female survived prolonged pulseless VT after three hours of continuous resuscitation and repeated defibrillation with bicarbonate therapy [14], aligning with our patient’s full recovery despite recurrent cardiac arrest.

## Conclusion

This case describes the clinical course and successful management of a single patient with severe amitriptyline poisoning complicated by multiple cardiac arrests in a resource-limited setting. It suggests that prolonged resuscitation could be continued in TCA-induced cardiac arrest, as favorable neurological outcomes are likely to occur. The report also highlights the importance of making life-saving therapies, such as sodium bicarbonate and lipid emulsion, readily available in hospitals. Serum alkalinization with intravenous bicarbonate remains the standard of treatment, in achieving cardiovascular stability and preventing fatal arrhythmias.

## Data Availability

No datasets were generated or analysed during the current study.

## Abbrevations

TCA: Tricyclic antidepressant
ACLS: Advanced cardiac life support
ROSC: Return of spontaneous circulation
CPR: Cardio pulmonary resuscitation
ICU: Intensive care unit
GABA: Gamma amino butyric acid
GCS: Glasgow coma scale
ECG: Electrocardiography
